# Demographically informed models for improving synthetic haematocrit and extracellular volume estimation in cardiac computed tomography

**DOI:** 10.1093/ehjimp/qyag020

**Published:** 2026-02-27

**Authors:** Sri Kousthubha Allampalli, Vitaliy Androshchuk, Edouard Long, Iulia Nazarov, Daniel Hodson, Tiffany Patterson, Simon Redwood, Ronak Rajani, Martin Bishop, John Whitaker

**Affiliations:** School of Biomedical Engineering and Imaging Sciences, King’s College London, Strand, London WC2R 2LS, UK; Department of Cardiology, Guy’s and St Thomas’ NHS Foundation Trust, Westminster Bridge Road, London SE1 7EH, UK; School of Cardiovascular Medicine and Sciences, Faculty of Life Sciences and Medicine, King’s College London, Strand, London WC2R 2LS, UK; Department of Cardiology, Guy’s and St Thomas’ NHS Foundation Trust, Westminster Bridge Road, London SE1 7EH, UK; School of Cardiovascular Medicine and Sciences, Faculty of Life Sciences and Medicine, King’s College London, Strand, London WC2R 2LS, UK; School of Biomedical Engineering and Imaging Sciences, King’s College London, Strand, London WC2R 2LS, UK; CT Practice and Development, Guy’s and St Thomas’ NHS Foundation Trust, Westminster Bridge Road, London SE1 7EH, UK; Department of Cardiology, Guy’s and St Thomas’ NHS Foundation Trust, Westminster Bridge Road, London SE1 7EH, UK; School of Cardiovascular Medicine and Sciences, Faculty of Life Sciences and Medicine, King’s College London, Strand, London WC2R 2LS, UK; Department of Cardiology, Guy’s and St Thomas’ NHS Foundation Trust, Westminster Bridge Road, London SE1 7EH, UK; School of Cardiovascular Medicine and Sciences, Faculty of Life Sciences and Medicine, King’s College London, Strand, London WC2R 2LS, UK; School of Biomedical Engineering and Imaging Sciences, King’s College London, Strand, London WC2R 2LS, UK; Department of Cardiology, Guy’s and St Thomas’ NHS Foundation Trust, Westminster Bridge Road, London SE1 7EH, UK; School of Biomedical Engineering and Imaging Sciences, King’s College London, Strand, London WC2R 2LS, UK; School of Biomedical Engineering and Imaging Sciences, King’s College London, Strand, London WC2R 2LS, UK; Department of Cardiology, Guy’s and St Thomas’ NHS Foundation Trust, Westminster Bridge Road, London SE1 7EH, UK

**Keywords:** synthetic haematocrit, extracellular volume, cardiac computed tomography, sex stratification, BMI stratification

## Abstract

**Aims:**

Cardiac computed tomography-derived extracellular volume (CCT-ECV) is a promising biomarker for non-invasive quantification of myocardial fibrosis. However, serum haematocrit (Hct) is required for accurate CCT-ECV calculation, posing a potential barrier to clinical implementation. This study aims to develop a method for predicting synthetic Hct to derive accurate ECV values without blood testing and investigate the impact of clinical factors on model performance.

**Methods and results:**

A total of 108 patients [70% male, body mass index (BMI) 27.2 (7.4) kg/m^2^, age 81.9 (8.6) years] undergoing CCT prior to clinically indicated transcatheter aortic valve implantation for severe aortic stenosis were recruited. A non-contrast baseline scan, electrocardiogram (ECG)-gated CT angiography, and a late iodine-enhanced scan were performed on the same day as blood tests for serum Hct and used to compute voxel-wise ECV in the left ventricle. A univariable linear regression model was developed to predict Hct from Hounsfield units at the centre of the blood pool, outperforming previous models in literature. Sex stratification improved accuracy, with a significant difference in models for men at a BMI threshold of 30.7 (*P* = 0.035). In females, restricting to BMI > 22.4 improved performance. Age, estimated glomerular filtration rate, and creatinine did not improve predictions. The final model with combined sex and BMI stratification demonstrated better performance (ECV Pearson *R* 0.89, *P* < 0.001) than univariable and literature models.

**Conclusion:**

This study highlights the necessity for sex-specific models to estimate Hct and accurately estimate ECV from CCT. Sex-specific BMI stratification further improves predictions; however, more research is required for females with a low or very high BMI.

## Graphical Abstract

**Graphical Abstract:**
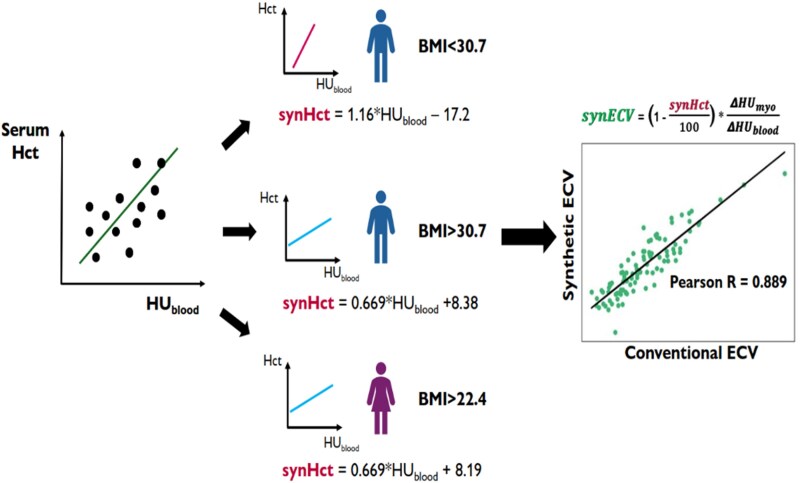
Trends between left ventricular blood pool Hounsfield units (HU_blood_) and serum haematocrit (Hct) were stratified by sex and body mass index (BMI), obtaining three separate regressions to optimize synthetic Hct (synHct) and extracellular volume (synECV) predictions.

## Introduction

Extracellular volume (ECV) describes the proportion of myocardium which is composed of non-cellular components such as extracellular fluid and collagen. ECV is increased in conditions that result in myocardial fibrosis, a common final pathway of adverse myocardial remodelling. It is conventionally measured using cardiovascular magnetic resonance (CMR) imaging which has been effectively used to demonstrate ECV expansion in conditions such as amyloidosis, myocarditis, and coronary artery disease,^1^ and therefore, increased ECV represents an important imaging biomarker of myocardial fibrosis.^2,3^ CMR-derived ECV has the advantage over other CMR-derived parametric mapping techniques, such as T_1_ mapping, of being less sensitive to scanner, field strength, and acquisition parameters.^4,5^ CMR-derived ECV has been validated against histology in clinical^6,7^ and pre-clinical^8,9^ studies and has previously shown to better delineate diffuse fibrosis where thresholded late gadolinium-enhanced cardiac magnetic resonance imaging (the current reference standard non-invasive approach for myocardial tissue characterization) might fail.^2,3,10^

Cardiac computed tomography (CCT) has recently been proposed as a potential alternative to CMR-derived ECV, offering higher spatial resolution, reduced costs, shorter imaging times, and more widespread availability.^11^ Both methods require measurement of serum haematocrit (Hct) values as close to the time of the scan as possible, with recent reports indicating a significant impact of time delays between blood sampling and imaging on ECV accuracy.^12^ This poses a potential barrier in ECV research and its integration into routine clinical workflows.

Previous studies in literature have proposed methods to estimate Hct using blood pool (BP) intensities in pre-contrast CMR and CCT scans, bypassing the need for invasive blood sampling and thus supporting the automation and scalability of ECV computation in research and clinical practice.^13–17^ Linear models to predict these values have been computed previously using CMR scans,^14^ dual energy,^17^ and photon-counting^18,19^ CCT scans; however, accurate methods for computation using single-energy CCT scans (which are more widely available) are still required. Previous studies have also shown that sex may influence the regressions derived from CMR scans^14^; however, similar analysis has not yet been conducted for CCT scans. This study aims to develop a predictive model to estimate Hct based on Hounsfield unit (HU) measurements of the BP from single-energy CCT scans and to evaluate whether demographic factors, such as sex, age, body mass index (BMI), estimated glomerular filtration rate (eGFR), and creatinine, can improve the predictive capability of the models for Hct and ultimately obtain better predictions of CCT-ECV.

## Methods

All patients provided written informed consent, and the study was conducted in accordance with the Declaration of Helsinki and approved by the Research Ethics Committee (REC reference 23/LO/0331, IRAS ID 319698). A prospective study of adults (>18 years) with severe symptomatic aortic stenosis (AS), defined by a peak aortic jet velocity ≥4 m/s, a mean aortic valve gradient ≥40 mmHg and an aortic valve area (AVA) <1 cm^2^/indexed AVA ≤0.6 cm^2^/m^2^ (in accordance with European Society of Cardiology and the European Association for Cardio-Thoracic Surgery guidelines) and undergoing clinically indicated transcatheter aortic valve implantation (TAVI), were recruited between June 2023 and November 2024. Patients underwent CCT for TAVI planning on the day of recruitment, along with laboratory investigations, baseline measurements of clinical characteristics (e.g. BMI) and echocardiography to obtain measurements of left ventricular ejection fraction (LVEF), AVA, and aortic valve peak velocity (AVV_max_), according to British Society of Echocardiography reporting guidelines.^20^ Patients with moderate-to-severe valvular heart disease other than AS were excluded from the study. Patient scans with registration or segmentation errors were later excluded from analysis.

### CCT image acquisition

All CCT scans were acquired on a Siemens SOMATOM Force Dual Energy scanner (Siemens Healthineers, Erlangen, Germany). A non-contrast baseline scan was obtained using a four-dimensional (4D) shuttle scan protocol at 80 kV for 15 s and with a slice thickness of 2 mm. A contrast agent (Omnipaque; GE Healthcare, Princeton, NJ, USA) was then administered with a dose of 100 mL at 5 mL/s followed by a 40 mL saline flush. A full retrospective electrocardiogram (ECG)-gated CT angiography (CTA) scan was triggered using bolus tracking in the proximal descending aorta following a 6 s delay once a HU threshold of 110 was reached and performed at 100 kV with a slice thickness of 1 mm. This was then followed by a FLASH CT aortogram for TAVI access planning. A late iodine-enhanced (LIE) 4D shuttle mode scan was performed at 5–7 min post-contrast at 80 kV for 15 s. The retrospective ECG-gated scan was reconstructed at every 5% of the *R–R* interval as well as at 250 ms. The baseline and late enhancement scans were both reconstructed during mid-systole at 250 ms.

### CCT image processing

**Figure 1:**
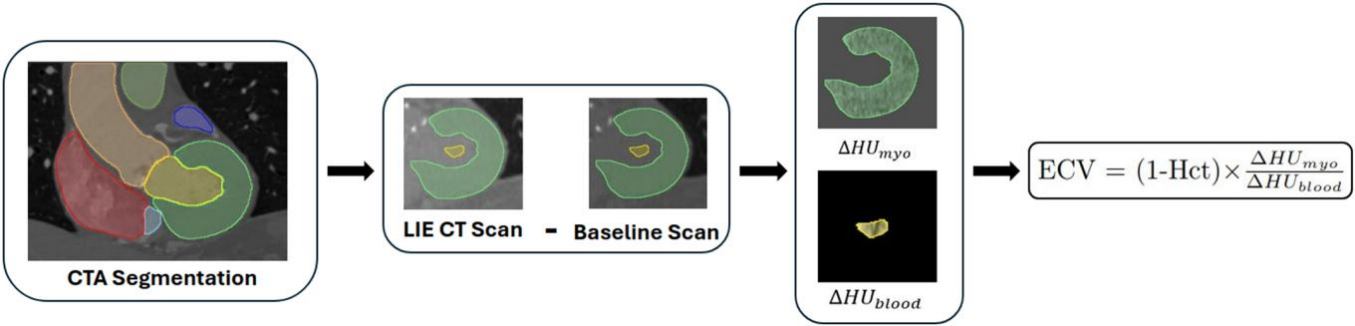
Image processing pipeline used to obtain ECV values from CCT scans.

Baseline and LIE scans were registered to the CTA scan using 3DSlicer.^21,22^ Left ventricle (LV) myocardium and BP masks were generated from the CTA image using NumeriCor’s CardioTwin/Studio software^23^ (NumeriCor GmbH, Graz, Austria). The segmentation was adjusted to enclose the papillary muscles within the BP mask. The BP mask was then eroded to only sample a small region at the centre of the LV to reduce the effect of errors in registration and minimize enclosing spurious HUs from trabeculations and metal artefacts. BP and LV masks were applied onto the baseline and LIE scans and the HUs from both were subtracted (*Figure 1*).

Serum Hct values were obtained from blood tests performed on patients on the same day as the CCT scans. These values were used to compute the conventional ECV values (conECV) in each voxel using the following equation:


(1) $$\mathrm{ECV}\;=\;(1-\mathrm{Hct})\;\times \;\frac{\Delta HU_{\mathrm{myo}}}{\Delta HU_{\mathrm{blood}}},$$

where ΔHU_myo_ is the change in the HUs in the LV myocardium in each voxel, and ΔHU_blood_ is the change in average HUs in the BP sample.

### Synthetic Hct and synthetic ECV

To estimate synthetic Hct values (synHct), linear regression models were fitted between the average HUs in the BP obtained from the baseline scans (HU_blood_) and serum Hct. A previous study by Treibel *et al.*^13^ derived a relationship between HU_blood_ and Hct with the following equation:


(2) $$\mathrm{synHct}=0.51\;\times HU_{\mathrm{blood}}+17.4.$$

**Figure 2:**
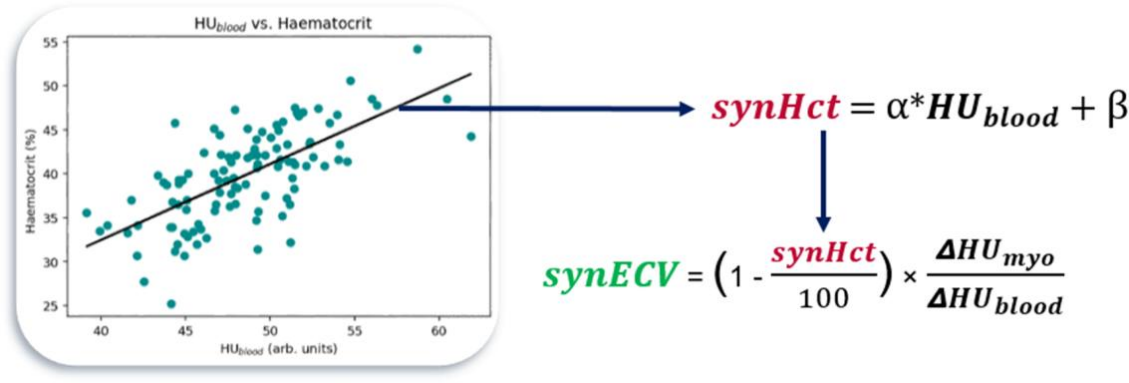
Diagram depicting the process for computing synHct and synECV.

Using an ordinary least squares linear regression method, a new model was fitted to the data and predictive capabilities of both models were compared. With the derived linear models, synHct values were calculated for each patient, and these values were then used to compute synthetic ECV (synECV) in each voxel by replacing the serum Hct in Eq. (1) with synHct (*Figure 2*). Average ECV across the LV was computed for each patient to compare between conECV and synECV results.

BMI, age, sex, eGFR, and creatinine were then included as interaction terms in the new linear model to assess their influence on the relationship between HU_blood_ and serum Hct. Age, BMI, eGFR, and creatinine were left as continuous variables, whereas sex was converted into a binary variable (male = 0, female = 1) and incorporated into the model as an interaction term, represented by the equation:


(3) $$\mathrm{synHct}=\alpha +\beta 1\times HU_{\mathrm{blood}}+\beta 2\times \mathrm{Sex}+\beta 3\times (HU_{\mathrm{blood}}\times \mathrm{Sex}).$$

To identify optimal bins for continuous variables, the dataset was divided into bins of two to five with varying widths, and predictions were computed for each. Only configurations with statistically significant *P* values for correlations in each bin were retained. Bin numbers were saved as categorical variables and included as interaction terms in the linear regression to assess whether the presence of these specific bins had an influence on the relationship between Hct and HU_blood_. The optimal bin configuration was chosen based on the highest combined mean absolute error (MAE) and Pearson *R* scores for Hct and ECV. If no bin configuration showed significant results, a sliding window of variable widths was applied across the range of values to identify the subset within which a statistically significant improvement in predictions was obtained.

### Statistical methods

Normally distributed variables are presented as mean ± standard deviation, whereas non-normally distributed variables are presented as median (interquartile range). Correlation coefficients between actual and synthetic Hct as well as conECV and synECV were calculated using Pearson *R*. MAE was used to determine the difference in values between actual and synthetic ECV and Hct. Linear model fitting was assessed by *R*^2^. *P* values of interaction terms were used to determine whether the influence of demographic factors on the relationship between HU_blood_ and Hct was significant.

## Results

### Patient characteristics

A total of 114 patients were included in the initial analysis. As 6 patients were excluded due to registration errors, this resulted in a final population of 108 patients, of which 75 were male and 33 were female. Patients recruited had severe symptomatic AS with a median AVA of 0.6 (0.3) and a median AVV_max_ of 4.25 (0.6). The cohort had a median BMI of 27.2 (7.4) kg/m^2^ and a median age of 81.9 (8.6) years. Patients exhibited a mean Hct of 0.40 ± 0.05 and median ECV of 0.32 (0.05). Other characteristics, stratified by sex, are presented in *Table 1*.

**Table 1:** Table presenting patient characteristics at the time of study

Patient characteristics	Male (*n* = 75)	Female (*n* = 33)
Age (years)	80 (10)	83 (7.5)
Height (m)	1.72 ± 0.1	1.58 (0.11)
Weight (kg)	79.0 (25)	63.0 (15)
BMI (kg/m^2^)	27.6 (7.7)	25.9 (7.3)
BSA (m^2^)	1.92 (0.3)	1.67 ± 0.2
Hypertension	21	8
Hypercholesterolaemia	29	10
Diabetes	0	0
Current or ex-smoker	0	0
Atrial fibrillation	52	30
Coronary artery disease	61	32
LVEF (%)	59.9 (8)	62.2 (10)
Aortic valve area (cm^2^)	0.7 (0.2)	0.6 (0.2)
Aortic valve max. velocity (m/s)	4.22 ± 0.5	4.46 ± 0.7
Haematocrit	0.402 ± 0.1	0.387 ± 0.04
Haemoglobin (g/L)	131 ± 19.4	126 ± 14.0
Creatinine (μmol/L)	95.0 (34)	77.0 (22.0)
eGFR (mL/min/1.73 m^2^)	62.7 ± 20.3	63.4 ± 17.9

### HU_blood_ vs. Hct univariable baseline model

A new linear model was fitted between HU_blood_ and Hct, obtaining the following equation:


(4) $$\mathrm{synHct}=0.864\;\times HU_{\mathrm{blood}}\;\text{–}2.09.$$

**Figure 3:**
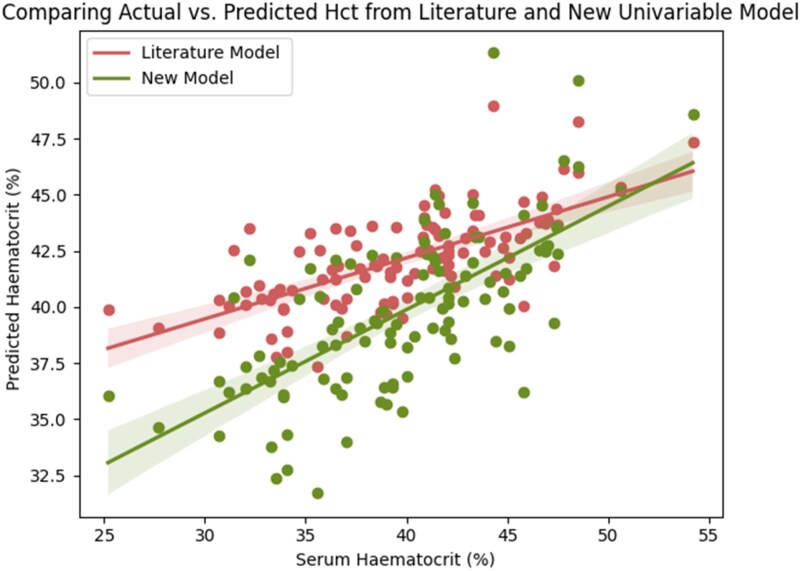
Graph comparing predicted Hct from the literature model and the new univariate model (fitted in this study) against serum Hct values.

The difference in Hct predictions between the Treibel model and this new model is presented graphically in *Figure 3*. When evaluating Hct prediction performance of each linear model (Eqs. 2 and 4), the Treibel model achieved an MAE of 0.04, whereas the newly derived model achieved an improved MAE of 0.03. For ECV estimation, the new model also resulted in a reduction in MAE from 0.02 to 0.017 and an improved Pearson *R* from 0.86 to 0.88 (*P* < 0.001).

### Influence of sex

**Figure 4:**
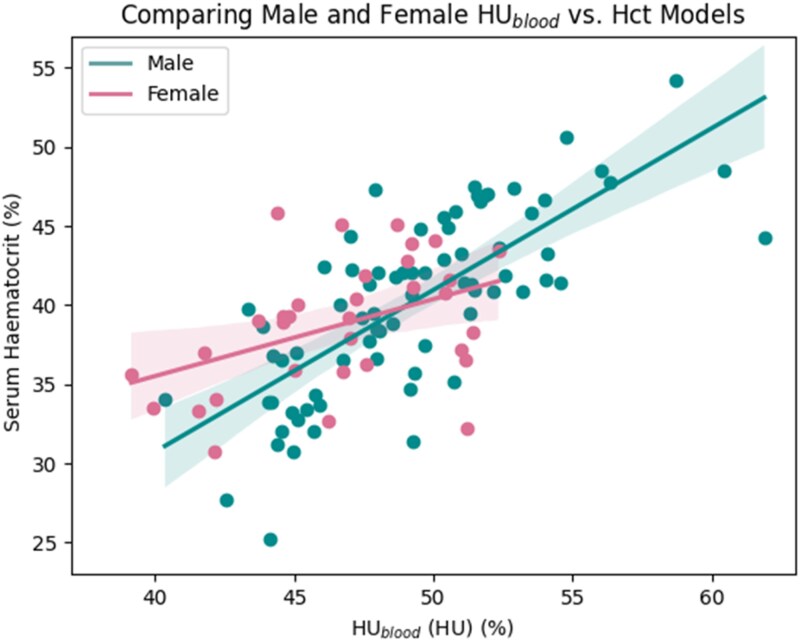
Graph comparing the difference in models between males and females.

Sex was found to have an influence on the linear relationship between HU_blood_ and Hct, indicated by a statistically significant *P* value of 0.015 for the HU_blood_ × sex interaction term (Eq. 3). The female model showed a sparser relationship compared with the male model, indicated by the lower *R*^2^ (0.18 compared with 0.55). The difference between the two models is shown in *Figure 4*. Having separate models reduced the MAE of both Hct and ECV (*Table 2*), and improved correlations to 0.70 and 0.90, respectively. All *P* values were statistically significant.

**Table 2:** Table summarizing performance metrics of each model.

	Literature model	Baseline model	Male model	Male model w/BMI	Female model	Female model w/BMI	Combined model
Hct mean absolute error	**0.037**	**0.031**	0.031	0.029	0.029	0.026	**0.030**
Hct Pearson *R*	**0.68** (95% CI: 0.56–0.77)	**0.68** (95% CI: 0.56–0.77)	0.74 (95% CI: 0.62–0.83)	0.77 (95% CI: 0.66–0.85)	0.43 (95% CI: 0.10–0.67)	0.57 (95% CI: 0.22–0.80)	**0.77** (95% CI: 0.68–0.84)
ECV mean absolute error	**0.020**	**0.017**	0.017	0.016	0.015	0.013	**0.016**
ECV Pearson *R*	**0.86** (95% CI: 0.80–0.90)	**0.88** (95% CI: 0.83–0.92)	0.89 (95% CI: 0.83–0.93)	0.90 (95% CI: 0.85–0.94)	0.86 (95% CI: 0.73–0.93)	0.86 (95% CI: 0.70–0.94)	**0.89** (95% CI: 0.84–0.93)

CI, confidence interval.

Bold values highlight the key models used for sequential comparison (literature model, newly-derived baseline model and final combined sex- and BMI-specific model).

### Influence of BMI

BMI as a continuous variable had a statistically significant influence on the baseline model only when applied to the male model. Binning BMI at a threshold of 30.7 for males further improved prediction metrics while also obtaining a significant *P* value for the interaction term of each bin (*P* = 0.035), validating the statistical significance of the BMI stratification on the baseline regression. Combining predictions from each bin-specific model improved Hct and ECV MAE compared with the male-only model without BMI (*Table 2*) and achieved an Hct Pearson *R* of 0.77 and ECV Pearson *R* of 0.90.

**Figure 5:**
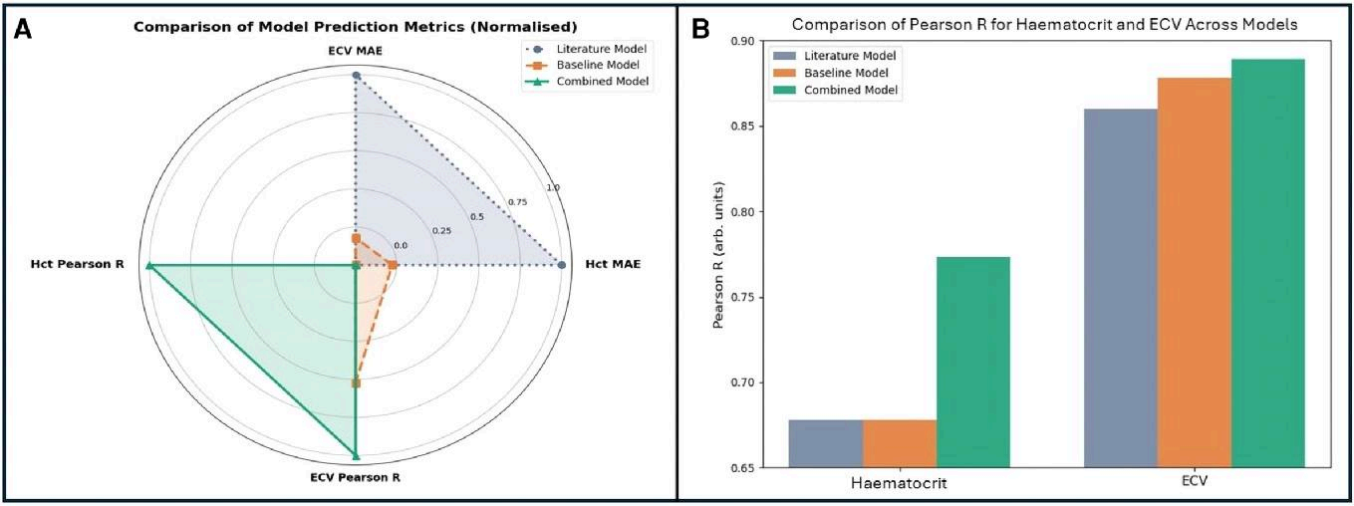
(*A*) Radar chart presenting the reduction in MAE and increase in Pearson *R* from the literature model (grey circle markers) to the baseline model (orange square markers) and finally to the combined sex- and BMI-specific model (green triangle markers). The values at each radius show the values of MAE and Pearson *R* normalized to the minimum and maximum results to highlight the relative improvement from each model. (*B*) Bar chart showing improvements in Pearson *R* from the baseline (middle orange bar) and combined model (rightmost green bar) compared with the literature model (leftmost grey bar).

In females, restricting the analysis to females with a BMI >22.4 resulted in improved model performance, increasing the *R*^2^ of the fit from 0.18 to 0.33. Within this subgroup, the Hct MAE and ECV MAE reduced (*Table 2*), and the Pearson *R* value for Hct increased to 0.57. The Pearson *R* for ECV did not change, obtaining a value of 0.86. All *P* values (for male and female BMI models) were statistically significant. Improvements in overall performance metrics by the addition of sex and BMI (compared with the literature and baseline models) are shown in *Figure 5A*.

### Influence of age, eGFR, and creatinine

Age, eGFR, and creatinine (as continuous variables) incorporated as interaction terms did not achieve statistically significant *P* values either for the combined sex model or for each sex separately. Binning also did not yield statistically significant *P* values for the interaction term of each bin for any of the variables.

### Sex- and BMI-specific model

Combining the different models according to sex and BMI resulted in an improved final model, utilizing the following sex- and BMI-specific regressions:


(5) $$\mathrm{Males},\;\mathrm{BMI}<30.7:\;\mathrm{synHct}=1.16\times HU_{\mathrm{blood}}\;\text{–}17.2$$

(6) $$\mathrm{Males},\;\mathrm{BMI}>30.7:\;\mathrm{synHct}=0.669\times HU_{\mathrm{blood}}+8.38$$

(7) $$\mathrm{Females},\;\mathrm{BMI}>22.4:\;\mathrm{synHct}=0.669\times HU_{\mathrm{blood}}+8.19$$

This model achieved an Hct MAE of 0.03 and ECV MAE of 0.016. Compared with both the literature model and the univariable model, the Pearson correlation coefficients improved from 0.68 to 0.77 (*P* < 0.001) for Hct, and from 0.86 and 0.88 to 0.89 (*P* < 0.001) for ECV.

## Discussion

This study reports a new method for the estimation of serum Hct from single-energy CCT imaging, including BMI- and sex-specific models which improve the diagnostic accuracy of both the serum Hct and CCT-derived ECV in this population.

Important findings are as follows:

1. A linear model derived from a larger cohort of patients than previous studies obtained better predictive capability of serum Hct compared with previous models reported in literature.
2. Patient sex significantly affects the relationship between HU_blood_ and Hct in single-energy CCT, motivating separate analysis.
3. Strength of relationships between HU_blood_ and Hct varied between males and females, and applying different BMI stratifications for each sex improved predictions of Hct and ECV.

### HU_blood_ vs. Hct univariable baseline model

Fitting a new linear model to the average HU_blood_ in the LV resulted in improved prediction metrics compared with the model reported in previous literature,^13^ with a significant *P* value for the correlation coefficient (*P* < 0.001). Specifically, the new model showed a reduction in MAE for both Hct and ECV, as well as an increase in the Pearson *R* for ECV (*Table 2*). The use of a larger cohort in this study (108 patients compared with 40) validates the improvement seen from the new linear model. However, studies have shown variability in blood HU for different tube voltages and scanner protocols, which may explain the disparity between these results.^24,25^

### Influence of sex

The interaction term HU_blood_ × sex obtained a significant *P*-value, indicating that the relationship between HU_blood_ and Hct does vary between males and females, thus warranting separate models for each sex. Additionally, the male model yielded a higher *R*^2^ compared with the female model, suggesting that the relationship is also not as well defined in females as it is in males and could be influenced by other factors. This further reinforces the idea that the relationship behaves differently between the sexes and therefore should be computed separately. These findings align with other studies that have computed separate models for each sex when predicting Hct using CMR scans.^14^

### Influence of BMI

The interaction term HU_blood_ × BMI yielded a significant *P*-value when applied only to the male model as opposed to the baseline (combined sex) model. This augments the necessity for separate models in predicting Hct.

Binning BMI into conventionally-defined categories^26^ showed evidence of improved correlations, however, did not yield significant *P* values for every bin. This motivated an investigation into determining an optimal bin size and number that obtained a statistically significant improvement to prediction metrics. Binning BMI in males by a threshold of 30.7 obtained the best performance metrics compared with the baseline model with combined sexes alone. Each bin yielded a significant *P* value and an improved *R*^2^, indicating that the improvement in metrics as a result of BMI binning is statistically significant and therefore presents the idea that the relationship between HU_blood_ and Hct varies in patients with higher BMIs, specifically those categorized as ‘Obese’.^26^ This could be attributed to the effect of a higher BMI on iron levels^27^ (as a high BMI can cause low-grade systemic inflammation which has in turn been shown to increase hepcidin and hence lower iron absorption^28^) and/or kidney function^29,30^ (e.g. because of haemodynamic changes from fat accumulation, hypertension, lower energy metabolism from low iron levels, etc.^31^). The different iron levels at different BMIs could translate to different relationships between the HUs and serum Hct, and previous studies have shown Hct prediction models breaking down in patients with anaemia.^27^ High BMIs have also been shown to impact image quality as the increased fat can increase noise in the image, and thus could affect the predictions from these images.^32^

Binning BMI in females did not have a significant effect in every bin; however, the *R*^2^ between HU_blood_ and Hct improved when the BMI range was restricted to BMI > 22.4 (along with improved prediction metrics). This suggests that a BMI that is too low could possess a different relationship between HU_blood_ and Hct; however, a significant linear relationship for patients in this range could not be obtained with the data present in this study. Distributions of patient characteristics between females with BMI > 22.4 and BMI < 22.4 did not appear to be significantly different; therefore, a biological reasoning for the wide variability in data in females with a BMI < 22.4 could not be derived from the data available in this study. Only two patients had a BMI > 35; therefore, there may also be a difference in relationship for patients with very high BMIs as well; however, there is not enough data in this study to draw this conclusion.

The model derived for females with a BMI > 22.4 was very similar to that derived for males with a BMI > 30.7. This may again be attributed to the fat distribution in the body affecting image quality, as BMI can have a greater impact on the levels of visceral fat in post-menopausal women compared with men,^33^ which could explain why a similar prediction model is observed in women at a lower BMI than men. Additionally, as breast tissue is predominantly composed of fat, this could further contribute to the reduced image quality if not accounted for appropriately. This would also support the reasoning for why similar models are observed at a lower BMI in women compared with men, and potentially offer an explanation for the different sex models. As visceral fat is not the only contributor to BMI, a high variability in body composition in the subset of females with a BMI < 22.4 may explain the lack of specificity in those results; however, this could not be conclusively confirmed from the patient characteristics available.

### Influence of age, eGFR, and creatinine

Incorporating age, eGFR, and creatinine as interaction terms in the linear model did not produce statistically significant results, whether treated as a continuous variable or categorized into bins, in either the combined sex model or the sex-specific models. Previous studies have shown an effect of age on synthetic CMR-ECV predictions^34^; however, because of the limited age distribution within this cohort of patients with severe AS [81.9 (8.6) years], a significant influence of age was not apparent. Renal function has been previously shown to directly correlate with Hct,^35^ with studies reporting a greater decline in Hct with worsening renal function in men compared with women among patients with impaired renal function.^36^ However, as calcification and progression of AS have been shown to be associated with kidney function,^37–39^ the use of a cohort of severe AS patients may have restricted the variation in renal function within each sex-specific subgroup, limiting the ability of the regression models to capture its potential influence. Analysis using a cohort with a broader range of ages and kidney function would be required to conclusively evaluate their influence.

### Combined sex- and BMI-specific model

By computing predictions using each sex- and BMI-specific model, prediction metrics for Hct and ECV improved from the baseline model that did not account for sex and BMI, and as these stratifications had significant *P* values for the interaction terms with the HU_blood_ vs. Hct regression model, this validated that the factors do in fact influence the accuracy in predictions for synthetically deriving Hct and ECV values in clinical practice. Improvements in Pearson *R* from the addition of sex and BMI stratification to the baseline model is presented in *Figure 5B*. This model, however, does not provide predictions for females with a BMI < 22.4 and may not fully represent the relationship in females with a BMI > 35.

The sex- and BMI-specific model yielded a significant improvement on the previously derived model in literature,^13^ and as the patient cohort was more than double the size, this further establishes the statistical significance of the improvement from the model. The trend in reduction of MAE and increase in Pearson *R* from the literature model to the baseline and finally to the combined sex- and BMI-specific model is highlighted in *Figure 5A*, underscoring the improvement on the literature model through the new linear fit and the role of sex and BMI stratification on further improvement of the predictions. These improvements, although incremental, contribute towards more accurate personalized ECV quantification, and highlights how sex and BMI should be considered when incorporating synthetic ECV prediction into clinical practice.

Leveraging these models for improved synthetic Hct prediction allows for the integration of ECV in myocardial characterization across a broad spectrum of cardiomyopathies, including cardiac amyloidosis, arrhythmia substrate identification, myocarditis, etc., without the need for invasive and time-consuming blood sampling. Personalized models that incorporate sex and BMI to improve ECV estimation may offer a reliable approach for incorporating ECV measurement into routine clinical practice and potentially help accelerate research into the clinical utility of ECV for various disease states.

### Limitations

The data for this study have been obtained from a single centre, and therefore, the regression models derived may be more applicable to the specific centre’s scanners and protocols. Although these results demonstrate that both sex and BMI should be accounted for in Hct predictions, the thresholds derived may be specific to this dataset, and therefore, a larger and more diverse cohort would be required to determine more generalized thresholds. Metal artefacts (present in eight patients) were also not removed from scans, and although efforts were taken to sample regions in the centre of the BP to minimize this error, there may still be artificially elevated voxels in these samples that could affect the models.

## Conclusion

In this study, it has been demonstrated that improved accuracy of synthetic Hct predictions can be achieved with the incorporation of sex and BMI as well as a refined linear regression equation. The relationship between HU_blood_ and Hct varies significantly between males and females, and the influence of BMI is sex-specific. The BMI- and sex-specific models reported resulted in significantly improved estimations of Hct and ECV compared with previously reported models and transition to clinical utilization of these models may improve diagnostic accuracy of CCT-derived ECV estimation, thus enabling a broader application of ECV for myocardial characterization in a spectrum of cardiomyopathies. Further research is required to evaluate the relationship between HU_blood_ and Hct in females with a low and high BMI as well as the mechanisms responsible for the observed differences.

## Data Availability

The data used in this study are available upon reasonable request with the corresponding author.

## Lead author biography

Sri Kousthubha Allampalli is a PhD student with the Digital Twins for Healthcare Centre for Doctoral Training at King’s College London. Her current research focuses on developing personalised cardiac digital twin models using novel imaging and computational methods to guide ventricular tachycardia ablation.
